# miR‐138 and miR‐193 target long non‐coding RNA UCA1 to inhibit cell proliferation, migration, and invasion of lung cancer

**DOI:** 10.1111/1759-7714.13605

**Published:** 2020-08-06

**Authors:** Guangsu Xun, Ming Ma, Bing Li, Song Zhao

**Affiliations:** ^1^ Department of Thoracic Surgery The First Affiliated Hospital of Zhengzhou University Zhengzhou China; ^2^ Department of Pharmacy The Second Affiliated Hospital of Henan University of Chinese Medicine Zhengzhou China

**Keywords:** Lung cancer, miR‐138, miR‐193, proliferation, UCA1

## Abstract

**Background:**

Long non‐coding RNA‐urothelial carcinoma associated 1 (LncRNA‐UCA1) is a crucial oncogene that is deregulated in many types of cancers. However, the mechanism of UCA1 function, especially for its posttranscriptional regulation in lung cancer, remains unclear.

**Methods:**

miRCode was used to predict potential miRNA candidates that might target UCA1. The targets of miR‐138 and miR‐193 on UCA1 and CDK6 were verified by luciferase reporter analysis. Western blotting was used to detect protein levels. The RNA level was evaluated using quantitative real‐time polymerase chain reaction (PCR). Proliferation, wound healing, and transwell invasion assays were performed to assess cell proliferation and invasion abilities. Correlations between miR‐138 or miR‐193 and UCA1 in lung cancer tissues was assessed using quantitative real‐time PCR.

**Results:**

miR‐138 and miR‐193 specifically targeted and regulated lncRNA‐UCA1. MiR‐138 and miR‐193 both suppressed cell proliferation and cell cycle progression. Moreover, miR‐138 and miR‐193 inhibited cell migration and invasion. Overexpression of UCA1 reversed the proliferation, migration, and invasion suppression effects of miR‐138 or miR‐193. Furthermore, miR‐138/193 affected the expression of UCA1 downstream genes. UCA1 regulated the expression of CDK6 as a miR‐138 and miR‐193 common target. In human lung cancer tissues, our study showed a significant negative correlation between miR‐138 or miR‐193 and UCA1 in lung cancer tissues.

**Conclusions:**

Our results demonstrated that miR‐138 and miR‐193 affect cell function by directly targeting and regulating UCA1 in lung cancer.

## Graphical abstract

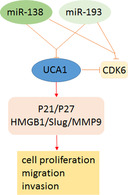

miR‐138 and miR‐193 both specially targeted and regulated lncRNA‐UCA1. Overexpression of UCA1 reversed the proliferation, migration and invasion suppression effect of miR‐138 or miR‐193. UCA1 regulated the expression of CDK6 as a miR‐138 and miR‐193 common target.

## Introduction

Lung cancer is a leading cause of death worldwide.^1^ In China, it is the leading cause of cancer‐related deaths.^2^ The management of lung cancer is a global health concern. Over the past decades, significant progress in the diagnosis and management of lung cancer has been made; however, the overall five‐year survival rate of lung cancer patients remains low.^3^ Therefore, understanding the molecular mechanisms that regulate the development and progression of lung cancer is necessary for improving its diagnosis and management.

Long non‐coding RNAs (lncRNAs) without protein coding (>200 nucleotides) play an important role in tumor progression.^4^, ^5^ LncRNAs act as oncogenes or tumor suppressors in various cancers.^6^ Urothelial carcinoma associated 1 (UCA1), an lncRNA, is reported to have an oncogenic function.^7^ The silencing of UCA1 significantly reduces the viability, migration, and invasion of hemangioendothelioma cells and induces apoptosis.^8^ UCA1 also increases the metastatic ability of gastric cancer cells,^9^ accelerates proliferation, increases cisplatin chemoresistance, and restrains apoptosis in oral squamous cell carcinoma cells.^10^

MicroRNAs (miRNAs), as a class of small endogenous non‐coding RNAs (approximately 19–25 nucleotides), bind to the 3′ untranslated regions (3′ UTRs) of target genes.^11^, ^12^ The aberrant expression of miRNAs can affect lung cancer progression.^13^, ^14^ MiR‐138 mainly acts as a tumor suppressor in various tumor types. MiR‐138 was reported to suppress low‐grade glioma development and metastasis.^15^ MiR‐138 suppresses cell proliferation and invasion in hepatocellular carcinoma.^16^ The aberrant expression of miR‐193 acts as a tumor suppressor in many types of tumors. MiR‐193 expression is significantly lower in colorectal cancer tissues than in the normal mucosa. The downregulation of miR‐193 is correlated with lymph node metastasis and poor survival of colorectal cancer patients.^17^ In pancreatic cancer, miR‐193 suppresses tumor growth and metastasis.^18^

Several studies have illustrated that lncRNA as a competitive RNA (ceRNA) for miRNA plays an important role in tumor development.^19^, ^20^ Therefore, we explored the interaction between lncRNA‐UCA1 and miR‐138 or miR‐193.

## Methods

### Cell culture

Cells were cultured in Dulbecco's modified Eagle's medium or 1640 medium (Gibco) containing 10% (v/v) fetal bovine serum (Gibco) at 37°C in a humid incubator with 5% CO_2_.

### Real‐time polymerase chain reaction (PCR)

The total RNA from tissues or cells was isolated using TRIzol (Invitrogen). RNA was reverse transcribed into cDNA with different reverse transcriptase primers using a reverse transcription kit (Applied Biosystems). PCR was performed using the TaqMan PCR kit (Applied Biosystems). Relative gene expression was calculated using the 2^−ΔΔCT^ method.

### Western blotting

Cells or tissues were collected and lysed with RIPA lysis buffer. After the samples were boiled, each cell lysate was separated by 10% sodium dodecyl sulfate polyacrylamide gel electrophoresis and transferred to polyvinylidene fluoride membranes (Millipore). The samples were incubated with the corresponding primary antibody and then with the secondary antibody. The proteins were visualized with enhanced chemiluminescence (Millipore) following the manufacturer's instructions.

### Luciferase reporter assay

The 3′ UTR of UCA1 or CDK6 containing the potential miR‐138 or miR‐193 binding site was constructed in pGL3 vectors. These vectors were transfected into lung cancer cells. Then, 48 hours later, luciferase activities were tested using the dual luciferase assay kit (Promega), with Renilla luciferase activity as the normalization standard.

### Cell viability assay

Cell viability was examined using a cell counting kit‐8 (CCK‐8) (Dojindo Molecular Technologies) according to the manufacturer's protocol; and 5000 cells/well were seeded in 96‐well plates. At the corresponding time, after 10 μL of the CCK‐8 reagent was added into each well, the plate was incubated for two hours at 37°C. The absorbance of each well was examined at 450 nm using a reader.

### Cell migration and invasion assays

For cell migration, a wound healing assay was conducted. The lung cancer cells were wounded by scraping and incubated in the medium for 24 hours. The migrated distance of the lung cancer cells was visualized under a microscope. The relative migrated distance of the cells was measured by the distance of cell migration/the distance measured at hour 0.

The invasion of lung cancer cells was measured using a transwell assay with 8 μm chambers (Corning). The transwell chambers were coated with 100 μL BD Matrigel. A total of 200 μL of cells at a density of 2 × 10^5^ cells/mL in the fetal bovine serum‐free medium was then seeded into the upper chambers, and the lower chambers were filled with 600 μL of the complete medium. After incubation for 48 hours, the invasive cells were stained with crystal violet and counted.

### Flow cytometry analysis

For cell cycle detection, cells were fixed with 70% ethanol at 4°C for two hours, before being stained with 50 μg/mL propidium iodide (PI, Sigma‐Aldrich) at 4°C for 30 minutes. The cell cycle was analyzed using flow cytometry (BD Biosciences).

### Clinical tissues

From August 2015 to August 2019, 20 patients diagnosed with lung cancer were included in this study at the First Affiliated Hospital of the Zhengzhou University. Tumor tissues and adjacent normal tissues from patients were frozen in liquid nitrogen after resection. None of the patients received any chemotherapy or radiation treatment before surgery. All patients provided written informed consent, and this study was approved by the Ethics Committee at our local institution.

### Statistical analysis

Data are presented as mean ± standard deviation. All results were obtained from at least three independent experiments. The Student's *t*‐test was used to compare two groups of data, and the one‐way ANOVA was used to compare multiple groups (≥3). Spearman's correlation was used to test the significance of the association between genes. *P‐*values <0.05 were considered statistically significant.

## Results

### UCA1 was a direct target candidate of miR‐138 and miR‐193

**Figure 1:**
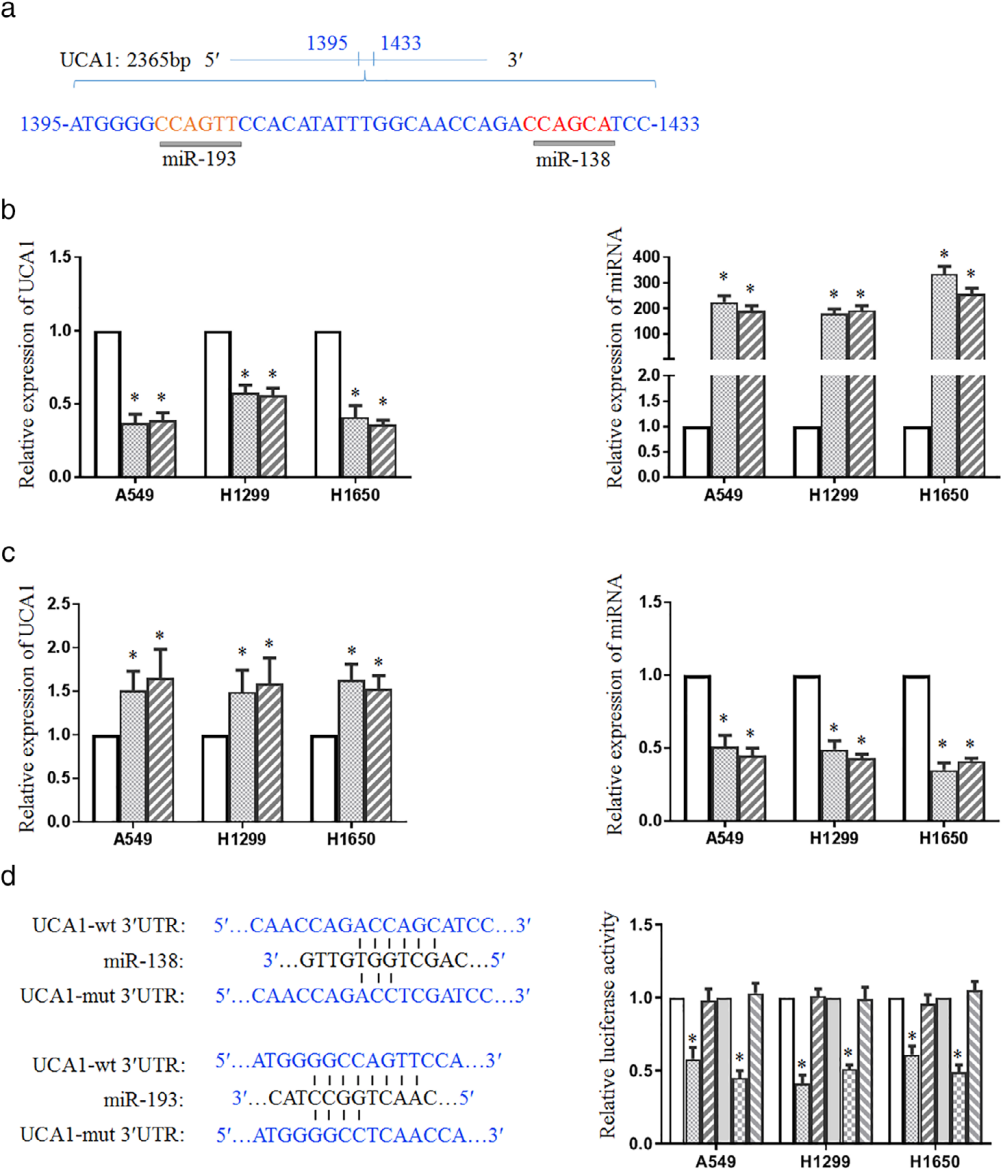
UCA1 is a direct target candidate of miR‐138/193. (**a**) The potential miRNAs targeting UCA1 in the region (1395–1433 bp) using bioinformatic analysis. (**b**) MiR‐138 and miR‐193 mimics were transfected into lung cancer cell lines (A549, H1299, and H1650). The RNA levels of UCA1 (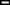) NC, (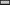) miR‐138, and (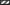) miR‐193 and miRNAs (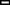) NC, (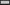) miR‐138, and (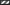) miR‐193 were tested using real‐time PCR. (**c**) The antagomirs of miR‐138 or miR‐193 were transfected into lung cancer cells and the corresponding RNA level was examined (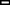) NC, (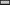) anti‐miR‐138, and (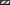) anti‐miR‐193; and (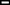) NC, (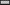) anti‐miR‐138, and (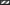) anti‐miR‐193. (**d**) Construction of wild‐ and mutant‐type binding sites between UCA1 and miR‐138 or miR‐193 in luciferase reporter vector was conducted. A549, H1299 and H1650 cells were cotransfected with UCA1 and wild‐type or mutant miR‐138 (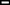) NC, (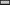) UCA1‐wt, and (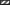) UCA1‐mut or miR‐193 (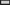) NC, (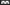) UCA1‐wt, and (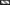) UCA1‐mut reporter vector, followed by the measurement of luciferase activities. *P < 0.05.

To predict potential miRNA candidates that might target UCA1, miRCode (www.miRCode.org) was used for analysis. The results showed that miR‐138/193 may be potential candidates binding to the region (1395–1433 bp) of UCA1 (Fig 1a). To test whether UCA1 was regulated by miR‐138/193, miR‐138 or miR‐193 mimics were transfected into lung cancer cell lines (A549, H1299, and H1650). As a result, the overexpression of miR‐138 or miR‐193 significantly decreased the expression level of UCA1 (Fig 1b). Furthermore, the application of antagomirs of miR‐138 or miR‐193 in lung cancer cells led to the enhancement of UCA1 RNA levels (Fig 1c). These results suggested that miR‐138 and miR‐193 could be potential miRNAs targeting UCA1. To examine whether miR‐138 or miR‐193 directly targets UCA1, we constructed luciferase reporter vectors that contained wild‐type or mutant UCA1 putative binding sites for miR‐138 or miR‐193 (Fig 1d). As shown in Figure 1d, the relative luciferase activity in cells transfected with UCA1‐3′ UTR‐wt for miR‐138 or miR‐193 was approximately 50% of the cells with negative control (NC) transfection. This luciferase activity was recovered when the potential miR‐138 or miR‐193 binding site was mutated. These results revealed that miR‐138 and miR‐193 directly targeted UCA1.

### UCA1 reversed the suppression effect of miR‐138/193 on cell growth, migration, and invasion

**Figure 2:**
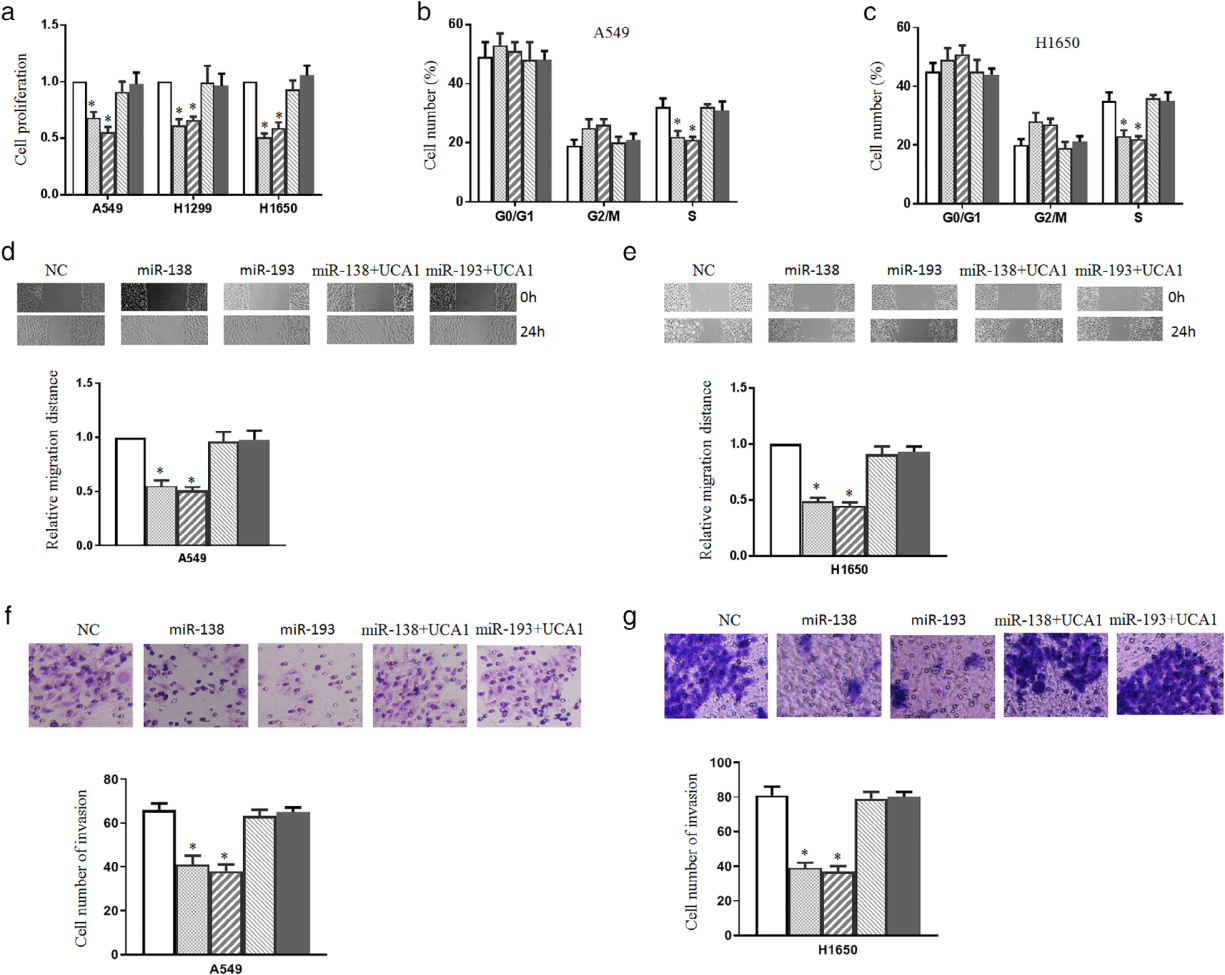
UCA1 reverses the suppression effect of miR‐138/193 on cell growth, migration, and invasion. (**a**) A549, H1299 and H1650 cells were transfected with miR‐138, miR‐193, miR‐138 plus UCA1 or miR‐193 plus UCA1, and cell proliferation was determined with a CKK‐8 assay (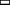) NC, (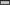) miR‐138, (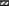) miR‐193, (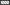) miR‐138+UCA1, and (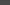) miR‐193+UCA1. (**b** and **c**) Cell cycle analysis was performed after transfection with miR‐138, miR‐193, miR‐138 plus UCA1 or miR‐193 plus UCA1 into A549 (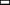) NC, (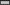) miR‐138, (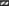) miR‐193, (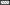) miR‐138+UCA1, and (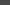) miR‐193+UCA1 and H1650 cells (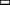) NC, (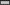) miR‐138, (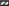) miR‐193, (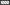) miR‐138+UCA1, and (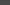) miR‐193+UCA1. (**d** and **e**) Wound healing assay was conducted after transfection with the corresponding vectors into A549 (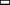) NC, (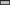) miR‐138, (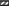) miR‐193, (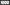) miR‐138+UCA1, and (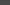) miR‐193+UCA1 and H1650 cells (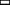) NC, (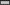) miR‐138, (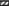) miR‐193, (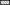) miR‐138+UCA1, and (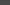) miR‐193+UCA1. The cell migration distance was measured. (**f** and **g**) The transwell assay was used to measure the cell invasion ability. A549 (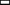) NC, (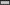) miR‐138, (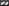) miR‐193, (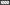) miR‐138+UCA1, and (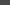) miR‐193+UCA1 and H1650 cells (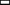) NC, (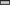) miR‐138, (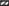) miR‐193, (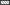) miR‐138+UCA1, and (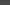) miR‐193+UCA1 were transfected with the indicated vectors. Cells on the lower surface of the chamber stained by crystal violet were counted. *P < 0.05.

To examine the effect of miR‐138 or miR‐193 on lung cancer cell growth, cell viability was assessed. The results showed that miR‐138 or miR‐193 significantly decreased the proliferation ability of A549, H1299, and H1650 cells (Fig 2a). In contrast, miR‐138 plus UCA1 or miR‐193 plus UCA1 recovered the cell growth ability. Cell cycle analysis was performed after transfection with miR‐138, miR‐193, miR‐138 plus UCA1, or miR‐193 plus UCA1. Flow cytometry showed that miR‐138 and miR‐193 significantly reduced the S phase population compared with the control group (Fig 2b,c). After UCA1 was added to the above vectors, the S phase population was reversed. In summary, these results indicated that UCA1 is a direct target of miR‐138 or miR‐193, responsible for suppressing cell proliferation and cell cycle progression.

Next, we examined the effect of miR‐138 or miR‐193 on lung cancer cell migration and invasion. Compared with the control group, the wound healing assay showed that miR‐138 and miR‐193 inhibited lung cancer cell migration after transfection with miR‐138 and miR‐193 (Fig 2d,e). UCA1 overexpression reversed the wound healing effect of miR‐138 or miR‐193. We observed that miR‐138 and miR‐193 also inhibited cell invasion (Fig 2f,g). UCA1 overexpression reversed miR‐138‐ or miR‐193‐induced invasion inhibition. These results indicated that UCA1 overexpression can overcome lung cancer cell growth, migration, and invasion suppression induced by miR‐138 or miR‐193.

### Effect of miR‐138/193 on UCA1 downstream genes

**Figure 3:**
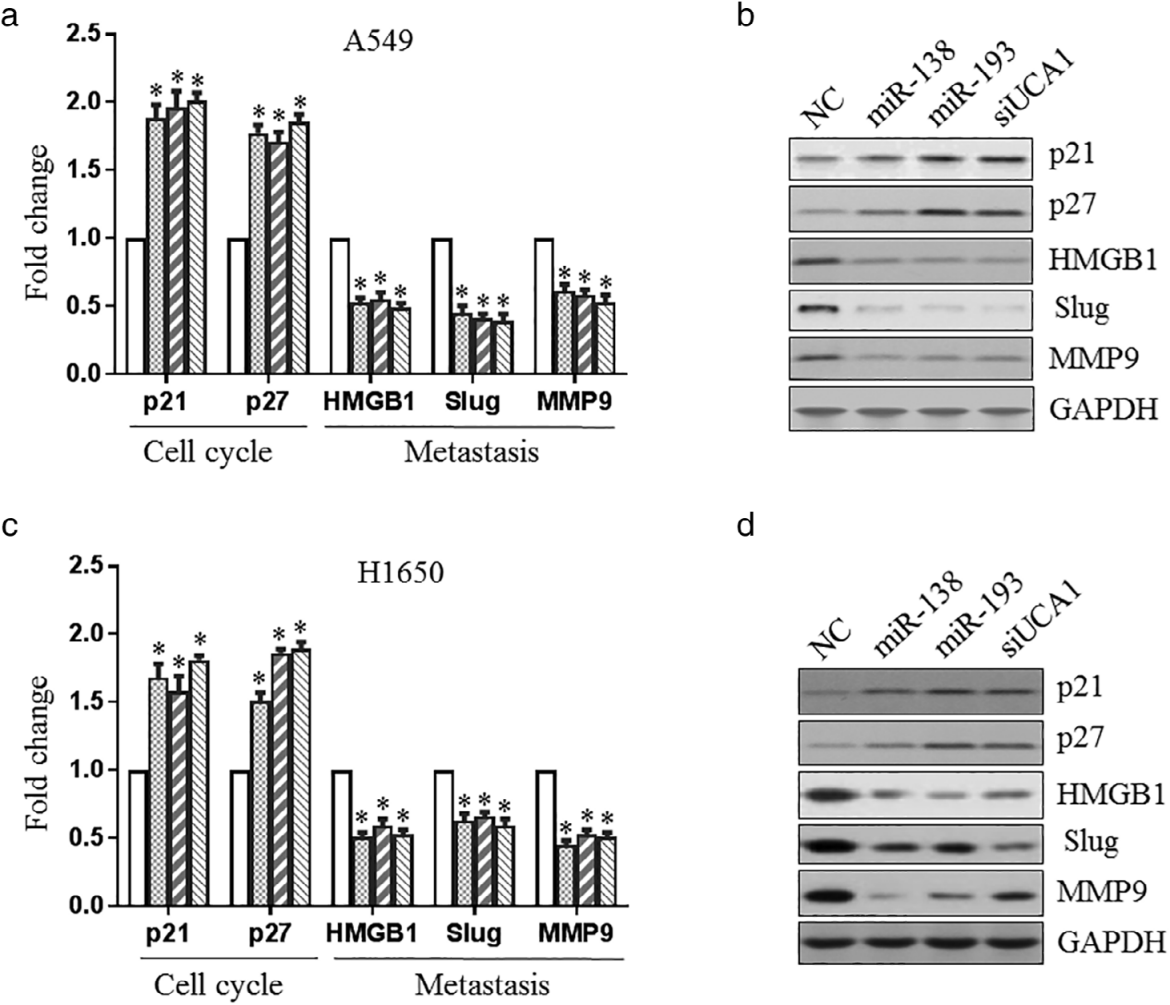
Effect of miR‐138/193 on UCA1‐downstream genes. A549 (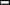) NC, (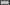) miR‐138, (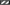) miR‐193, and (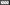) siUCA1 (**a** and **b**) and H1650 (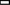) NC, (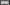) miR‐138, (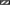) miR‐193, and (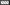) siUCA1 (**c** and **d**) cells were transfected with miR‐138, miR‐193 or UCA1 siRNA, before the UCA1 downstream genes related to cell cycle (p21 and p27), migration, and invasion (HMGB1, Slug and MMP9) were examined using real‐time PCR (**a** and **c**) or western blotting (**b** and **d**).

Given the effect of miR‐138 or miR‐193 on cell cycle, migration, and invasion, the UCA1 downstream genes related to the cell cycle (p21 and p27), migration, and invasion (HMGB1, Slug, and MMP9) were studied. First, we examined these genes in A549 cells and found that the overexpression of miR‐138/193 or knockdown of UCA1 promoted the mRNA expression levels of p21 and p27 and alleviated the mRNA expression levels of HMGB1, Slug, and MMP9 (Fig 3a). Western blotting showed that miR‐138, miR‐193, or UCA1 siRNA enhanced the levels of p21 and p27 and reduced the protein levels of HMGB1, Slug, and MMP9 (Fig 3b). This effect also occurred in H1650 cells (Fig3c,d). These results suggested that miR‐138 or miR‐193 regulates the cell function of UCA1 by affecting UCA1 downstream genes.

### UCA1 regulated the expression of CDK6 as a miR‐138 and miR‐193 target

**Figure 4:**
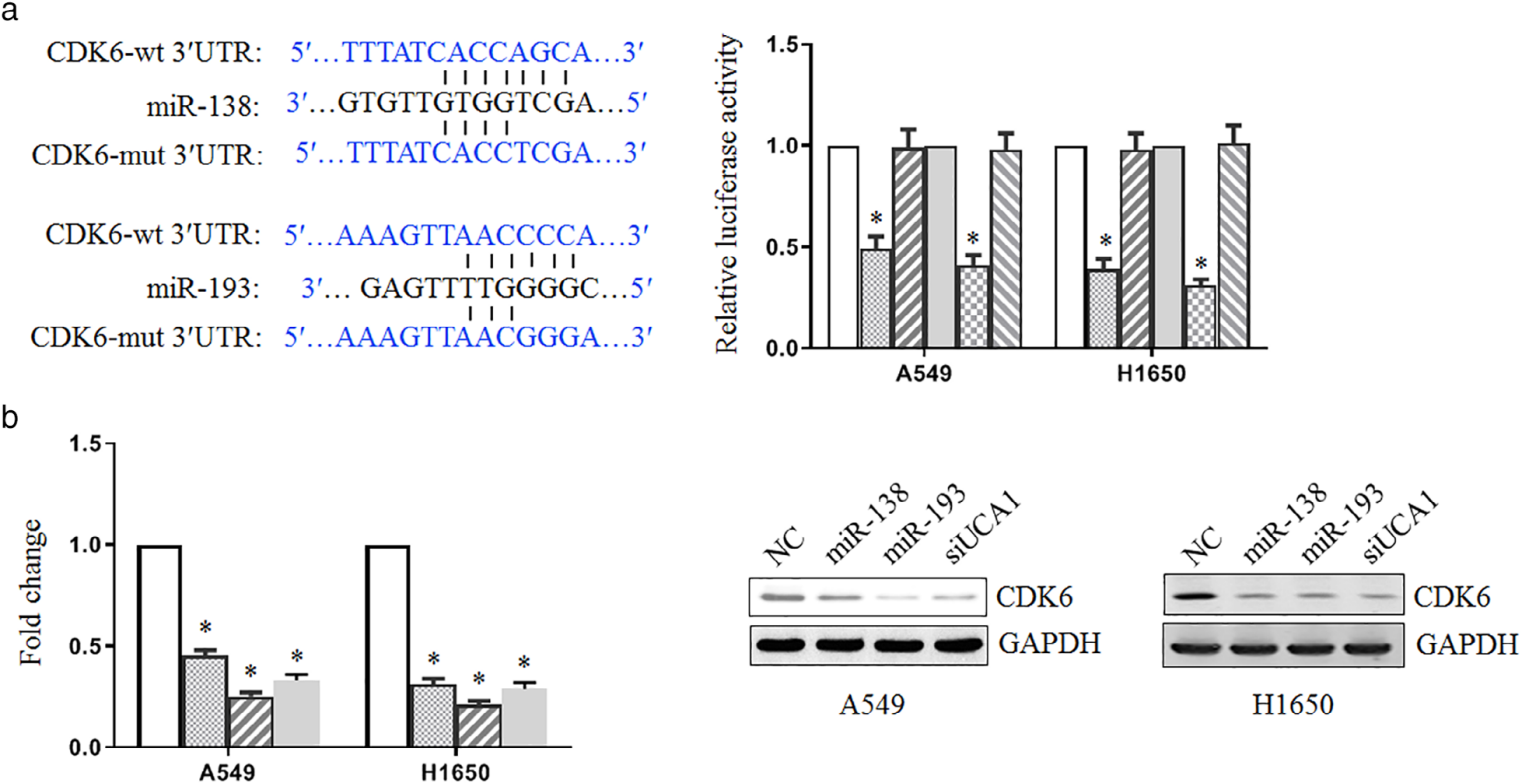
UCA1 regulates the expression of CDK6 as a miR‐138 and miR‐193 target. (**a**) The miR‐138 (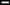) NC, (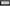) CDK6‐wt, and (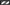) CDK6‐mut and miR‐193 (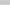) NC, (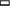) CDK6‐wt, and (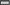) CDK6‐mut binding site predicted in the 3′ UTR of CDK6. A construct was also generated targeting the seed region of CDK6 3′ UTR. The luciferase activity was determined 48 hours after transfection with wild‐type or the mutant luciferase reporter vector. (**b**) The CDK6 mRNA level was examined using real‐time PCR (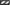) NC, (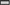) miR‐138, (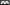) miR‐193, and (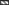) siUCA1. The CDK6 protein was examined using western blotting.

Since miR‐138/193 affected the expression of UCA1 downstream genes, we wondered whether UCA1 regulated the target genes of miR‐138 and miR‐193. First, we searched for genes targeted by miR‐138 and miR‐193 using the biological target prediction software, TargetScan. CDK6 harbored the potential miR‐138 or miR‐193 binding site within its 3′ UTR (Fig 4a). The wild‐type or mutant 3′ UTR sequence of CDK6 was cloned into a luciferase reporter vector to determine whether CDK6 is targeted by miR‐138 or miR‐193. The luciferase activity of the wild‐type construct was significantly decreased compared with that of the control in A549 and H1650 cells, whereas its mutant counterpart was not (Fig 4a). These results suggest that CDK6 is a direct target of both miR‐138 and miR‐193. In addition, the mRNA and protein levels of CDK6 in A549 and H1650 cells were dramatically reduced by miR‐138 and miR‐193 (Fig 4b). Moreover, the knockdown of UCA1 inhibited the mRNA and protein levels of CDK6. These results indicated that UCA1 might regulate the expression of CDK6 through miR‐138 and miR‐193.

### MiR‐138/193 are negatively correlated with UCA1

**Figure 5:**
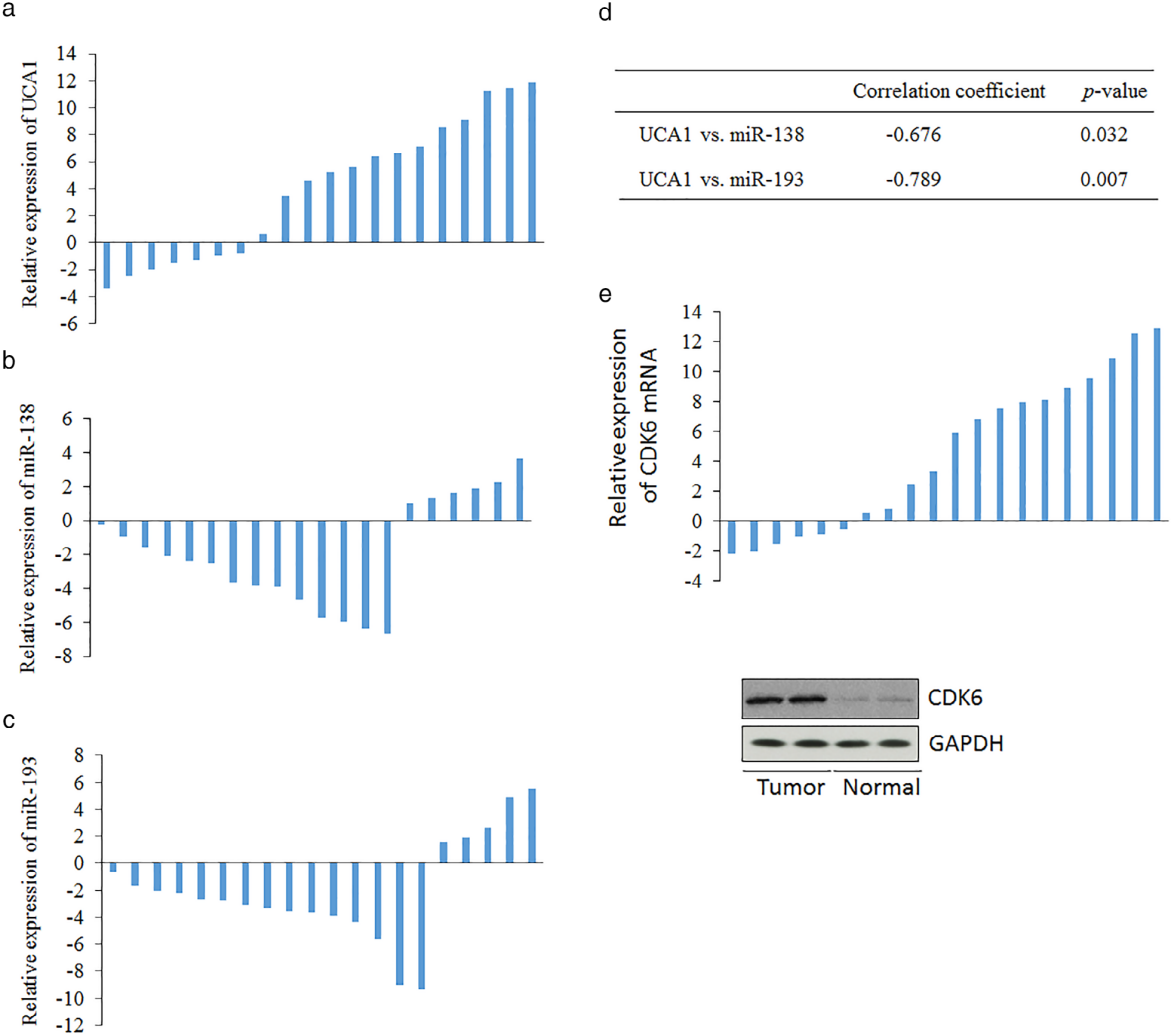
miR‐138/193 are negatively correlated with UCA1. (**a**) UCA RNA level was tested in lung cancer and normal tissues using real time‐PCR. RNA level of miR‐138 (**b**) or miR‐193 (**c**) was also detected in lung cancer and normal tissues by real‐time PCR. (**d**) The correlations between miR‐138 or miR‐193 and UCA1. The relative expression of the responding genes was counted by cancer/normal data.

Finally, the expression of miR‐138/193 and UCA1 was examined in lung cancer and adjacent normal tissues. PCR showed that UCA1 was highly expressed in lung cancer tissues compared with normal tissues (Fig 5a). However, a notable decrease in miR‐138 RNA levels was found to be higher in cancer tissues than in normal tissues (Fig 5b). In addition, the downregulation of miR‐193 expression in lung cancer tissues was confirmed (Fig 5c). Moreover, there was a significant negative correlation between miR‐138 and miR‐193 and UCA1 in lung cancer tissues (Fig 5d). We also found that CDK6 mRNA and protein levels were higher in lung cancer tissues than in normal tissues (Fig 5e).

## Discussion

The interaction between lncRNA and miRNA functional networks has recently been widely investigated in cancers. In colorectal cancer, lncRNA–CCAT2 as a negative regulator of miRNA‐145 biogenesis affects colon cancer stem cell proliferation and differentiation.^21^ LncRNA HAND2‐AS1 suppresses colorectal cancer progression by sponging miR‐1275 to modulate KLF14 expression.^22^ LncRNA XLOC_006390 as a ceRNA regulates the expression of miR‐331–3p and miR‐338‐3p to facilitate cervical cancer tumorigenesis and metastasis.^23^ In the present work, we demonstrated that miR‐138 and miR‐193 target UCA1. Studies on lung cancer biology showed that miR‐138 and miR‐193 suppressed cell proliferation and cell cycle progression. Moreover, we also found that miR‐138 and miR‐193 inhibited cell migration and invasion. Overexpression of UCA1 reversed the suppression effect of miR‐138 or miR‐193. Particularly, miR‐138/193 affected UCA1 downstream genes. In human lung cancer tissues, our results showed a significant negative correlation between miR‐138 or miR‐193, and UCA1 in lung cancer tissues.

LncRNA‐UCA1 plays a critical role as an oncogene in cancer. The study of the interaction between lncRNA‐UCA1 and miRNA mainly focuses on UCA1 targeting for miRNA. UCA1 targets miR‐107 to promote pancreatic cancer progression.^24^ UCA1 represses miR‐26a/b, miR‐193a, and miR‐214 expression through direct interaction in gastric cancer.^25^ UCA1 increases cell proliferation and multidrug resistance of retinoblastoma cells by downregulating miR‐513a‐5p.^26^ However, there have been few studies on miRNAs targeting UCA1. Most studies focus on one miRNA targeting one target gene in a single way. Every gene was targeted and regulated by multiple miRNAs. Therefore, in this study, the potential miRNAs targeting UCA1 were explored, and we found that miR‐138 and miR‐193 both targeted UCA1, and UCA1 function can be also regulated by miR‐138 or miR‐193.

MiR‐138 mainly suppresses cell proliferation and metastasis of cancers, including lung cancer. MiR‐138‐5p inhibits cell migration, invasion, and EMT in breast cancer.^27^ MiR‐138 inhibits the proliferation, invasion, and migration of ovarian cancer cells.^28^ MiR‐138 suppresses the proliferation, metastasis, and autophagy of non‐small cell lung cancer.^29^ Our results supported the above function of miR‐138: miR‐138 suppressed cell proliferation and invasion. The role of miR‐193 in lung cancer was also investigated. Our findings suggest that miR‐193 suppresses cell proliferation, migration, and invasion, which is consistent with the functions of miR‐193 in many tumors. For example, in prostate cancer, osteosarcoma, and hepatocellular carcinoma, miR‐193 impaired tumor cell growth and migration.^30^, ^31^, ^32^

Since miR‐138/193 affected the expression of UCA1‐downstream genes, we wondered whether UCA1 regulated the target genes of miR‐138 and miR‐193. We found that the knockdown of UCA1 inhibited the mRNA and protein levels of CDK6 as a common target of miR‐138 and miR‐193. These results indicated that UCA1 might regulate the expression of CDK6 through miR‐138 and miR‐193. CDK6 as an oncogene has been reported to be overexpressed in cancer cells.^33^ This is consistent with our results.

In conclusion, the results from the present study demonstrated that miR‐138 and miR‐193 as tumor suppressors targeted and regulated the oncogenic function of lncRNA‐UCA1. These findings will help us understand the interaction between miRNAs and lncRNA‐UCA1 in lung cancer.

## Disclosure

The authors declare that there are no conflicts of interest to report.
